# Undifferentiated pleomorphic sarcoma of the adrenal gland: a case report and literature review

**DOI:** 10.3389/fonc.2024.1439357

**Published:** 2024-11-18

**Authors:** Gong Xiaochuan, Zhao Wei, Yuan Chaoyong, Zhou Yu, Jian Huayong, Yin Na, Luo Xike, Lei Jian, Wang Yan

**Affiliations:** ^1^ Department of Urinary Surgery, Zunyi Medical University Third Affiliated Hospital the First People’s Hospital of Zunyi, Zunyi, China; ^2^ Department of Pathology, Zunyi Medical University Third Affiliated Hospital the First People’s Hospital of Zunyi, Zunyi, China

**Keywords:** adrenal gland, undifferentiated pleomorphic sarcoma, malignant fibrous histiocytoma, soft tissue sarcomas, chemotherapy

## Abstract

Undifferentiated pleomorphic sarcoma (UPS) is a rare type of tumor, and UPS originating in the adrenal gland is even rarer. Up to now, there have been no reports in English literature of UPS originating from the adrenal gland. This case report presents a 44-year-old female patient with UPS of the adrenal gland, who has shown no signs of recurrence or metastasis half a year after undergoing resection of a left adrenal tumor. A retrospective analysis of the patient’s diagnosis and treatment process is conducted, with the aim of providing a reference for the diagnosis and treatment of adrenal UPS.

## Introduction

Soft tissue sarcomas (STS) are rare malignant tumors that arise in the mesenchymal tissue, accounting for approximately 2% of adult malignancies (1). Undifferentiated pleomorphic sarcoma (UPS), formerly known as malignant fibrous histiocytoma (MFH), was renamed by the World Health Organization in 2013. It is an aggressive soft tissue sarcoma that constitutes 10-20% of STS cases (2). It is most commonly observed in the extremities and trunk. Although it is extremely rare for this tumor to occur outside these regions, there are documented cases of its occurrence in the head and neck, retroperitoneal space, and other visceral organs, including the spleen (3–5).

The incidence of this disease is low, with reports indicating 0.08 to 1 case per 100,000 people (6), hence there are no clear staging criteria for this tumor, nor are there definitive treatment guidelines. The main treatment methods currently include radical surgical resection, followed by chemotherapy or radiotherapy postoperatively based on tumor size and the presence of metastatic signs. Despite employing such aggressive treatment approaches, the 5-year recurrence rate of this disease ranges from 10% to 42%, and there is a 31% to 40% chance of distant metastasis; the 5-year survival rate also only lies between 30% to 50% (7).

Upon review of the literature, it is noted that, as of the writing of this report, there have been no cases of UPS originating in the adrenal gland reported in the literature, with only two cases documented as metastatic UPS to the adrenal gland (8, 9). Moreover, due to the rarity of this disease in the adrenal gland, it is often misdiagnosed as pheochromocytoma or other adrenal tumors. Therefore, a case of primary adrenal UPS is reported as follows.

## Case report

A 44-year-old female was admitted to the hospital due to “left-sided lumbar pain for 20 days.” The patient reported experiencing intermittent dull pain in her left waist without any apparent cause, without dizziness, headache, thirst, fatigue, or paresthesia in the extremities, and without urinary symptoms such as frequency, urgency, or pain. Physical examination revealed stable vital signs and normal blood pressure, with no signs of centripetal obesity or moon face indicative of increased cortisol levels. There was no swelling or prominence in the bilateral renal areas, no tenderness upon percussion in the bilateral renal regions, and no deep tenderness in the bilateral ureteral course. The patient had a history of hypertension for one year; there was no family history of hypertension.

**Figure 1:**
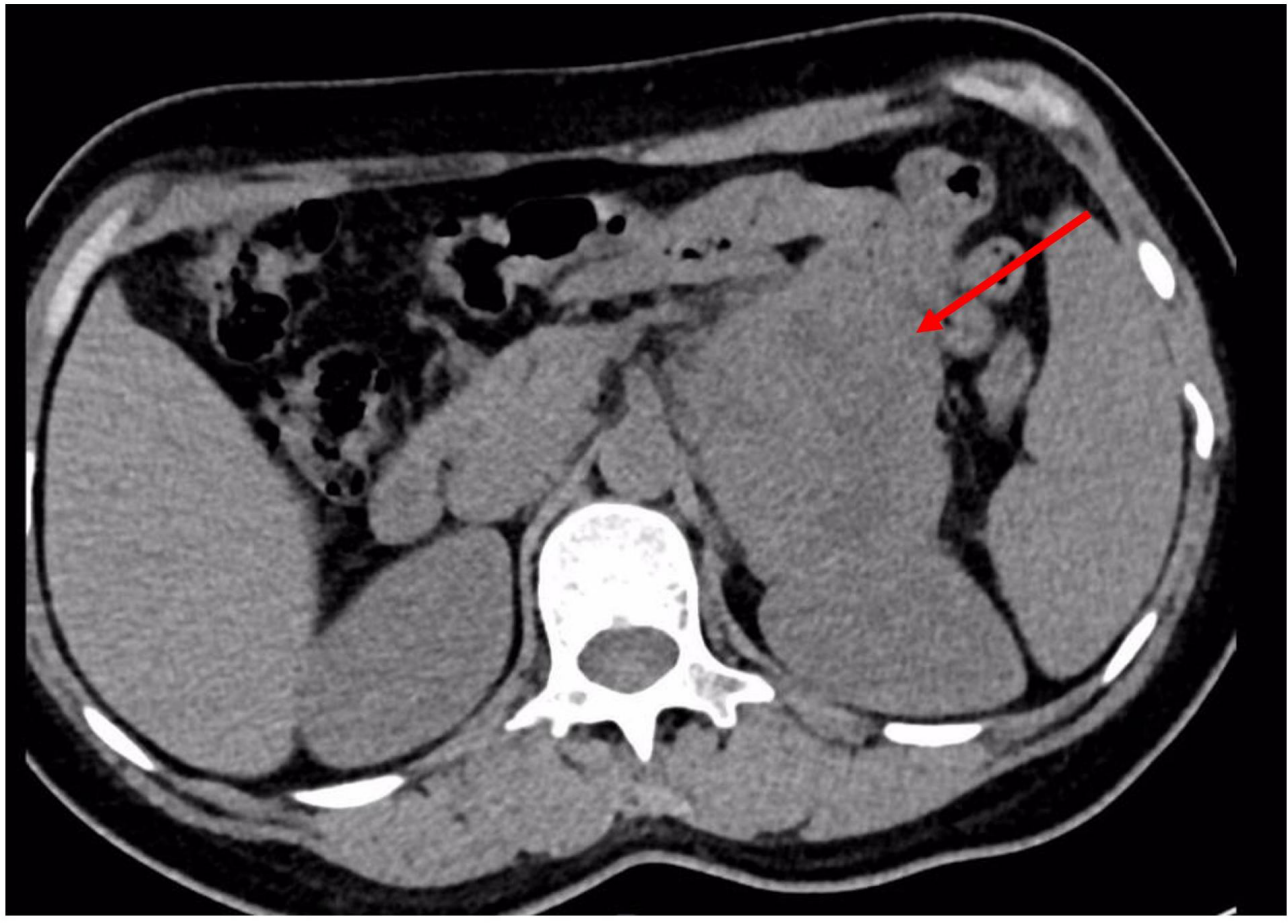
Abdominal CT reveals a heterogeneously dense mass in the left adrenal region, measuring approximately 7.9 cm × 6.1 cm.

**Figure 2:**
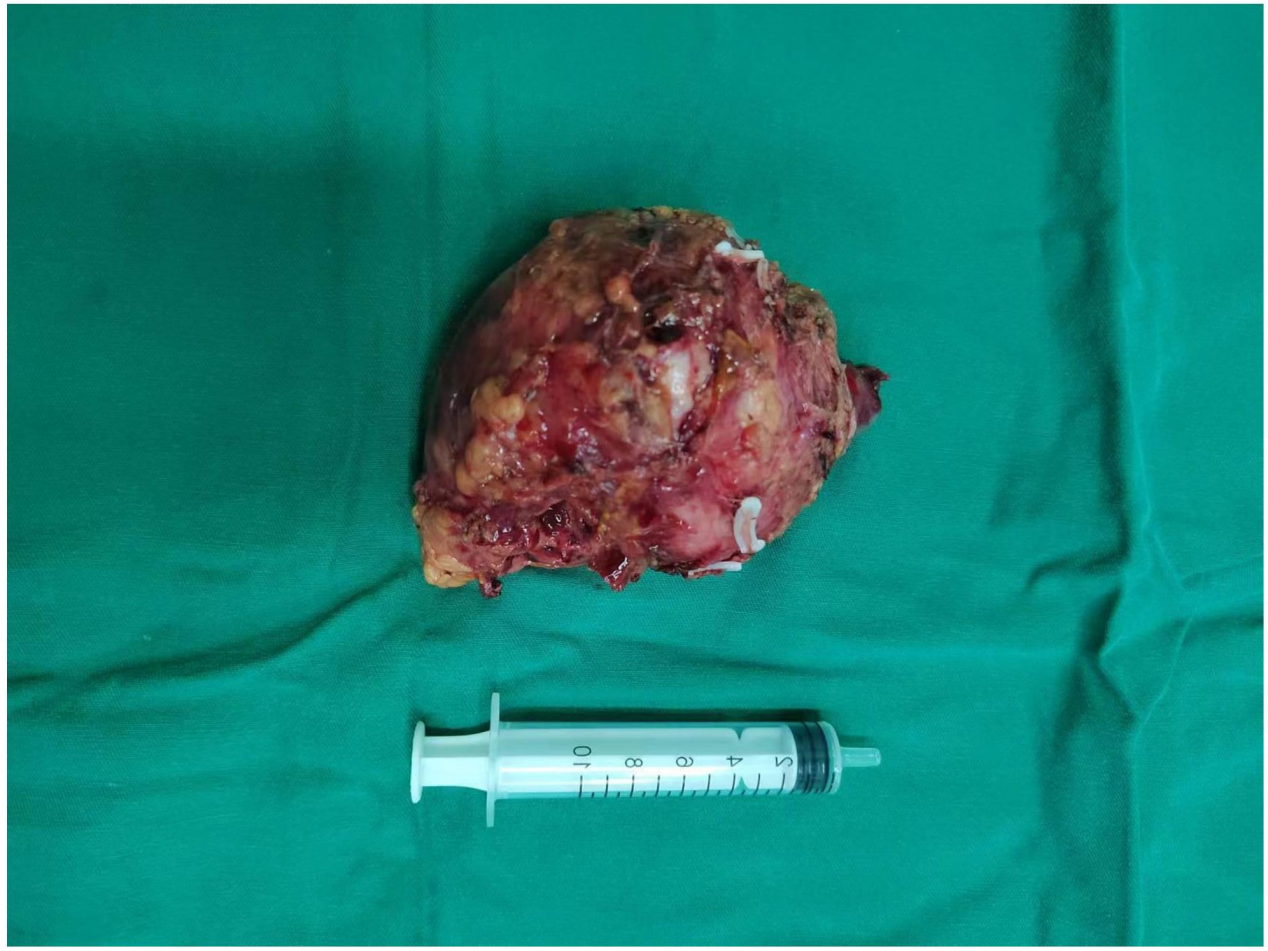
Tumor Tissue: The tumor tissue exhibits abundant blood supply and an intact capsule.

Chest and adrenal computed tomography (CT) scan revealed no significant abnormalities in the chest. A mass with mixed densities measuring approximately 7.9 cm × 6.1 cm was observed in the left adrenal gland, predominantly of soft tissue density, with irregular cystic areas within. Contrast-enhanced scanning showed significant heterogeneous enhancement of the solid portions, with smooth edges; the surrounding fat planes were clear, suggesting a high likelihood of pheochromocytoma (**Figure 1**). Whole-body PET/CT scan indicated no metastatic lesions or other primary lesions. Serum cortisol, angiotensin II, renin, aldosterone, and the aldosterone-renin ratio were all within normal limits. After completing all necessary examinations and following multidisciplinary discussion, the possibility of a pheochromocytoma was considered high, but the possibility of a malignant tumor was not excluded. With the consent of the patient and her family, a laparoscopic left adrenalectomy for tumor removal was performed. After the induction of general anesthesia, the patient was placed in a left lateral decubitus position. A longitudinal incision of approximately 4 cm was made at the level of the umbilicus along the lateral edge of the left rectus abdominis muscle. A 30° laparoscope was inserted. Under laparoscopic observation, puncture points were made 2 cm below the costal margin and at the level of the umbilicus along the left anterior axillary line, where 10 mm and 5 mm trocars were inserted. Pneumoperitoneum was established and maintained at a pressure of 12 mmHg. The abdominal cavity was inspected for any injury or bleeding. The lateral peritoneum was incised with an ultrasonic scalpel in the lateral paracolic gutter, and the descending colon was mobilized medially. The spleen’s lateral and superior peritoneum were further incised to expose the upper pole of the left kidney, where the adrenal tumor was visible superiorly and medially. Small branches of the diaphragmatic artery were dissected and ligated with an ultrasonic scalpel, bipolar coagulation, and synthetic clips, and the same technique was used to free the medial edge from small branches originating from the aorta and the lateral inferior branches between the left renal artery and vein. The central adrenal vein was exposed by mobilizing the adrenal gland along the upper side of the left renal vein, and after applying three synthetic clips, the central vein was transected. Finally, the lateral and posterior walls of the adrenal gland were freed. After completely mobilizing the left adrenal gland, the surgical field was carefully checked for any obvious bleeding. Intraoperative frozen section results: Tumor cells were identified with significant cellular atypia and pleomorphism, suggesting the diagnosis of undifferentiated pleomorphic sarcoma of the adrenal gland. The tumor was approximately 5 centimeters from the closest resection margin of the adrenal gland, and the surgical margins were negative. Therefore, Two abdominal drains, F24 and F16, were placed in the abdominal cavity, one at the upper pole of the left kidney and the other at the central vein transection site. Finally, after retracting the laparoscope, the incision was closed layer by layer (**Figure 2** for the gross appearance of the tumor postoperatively).

**Figure 3:**
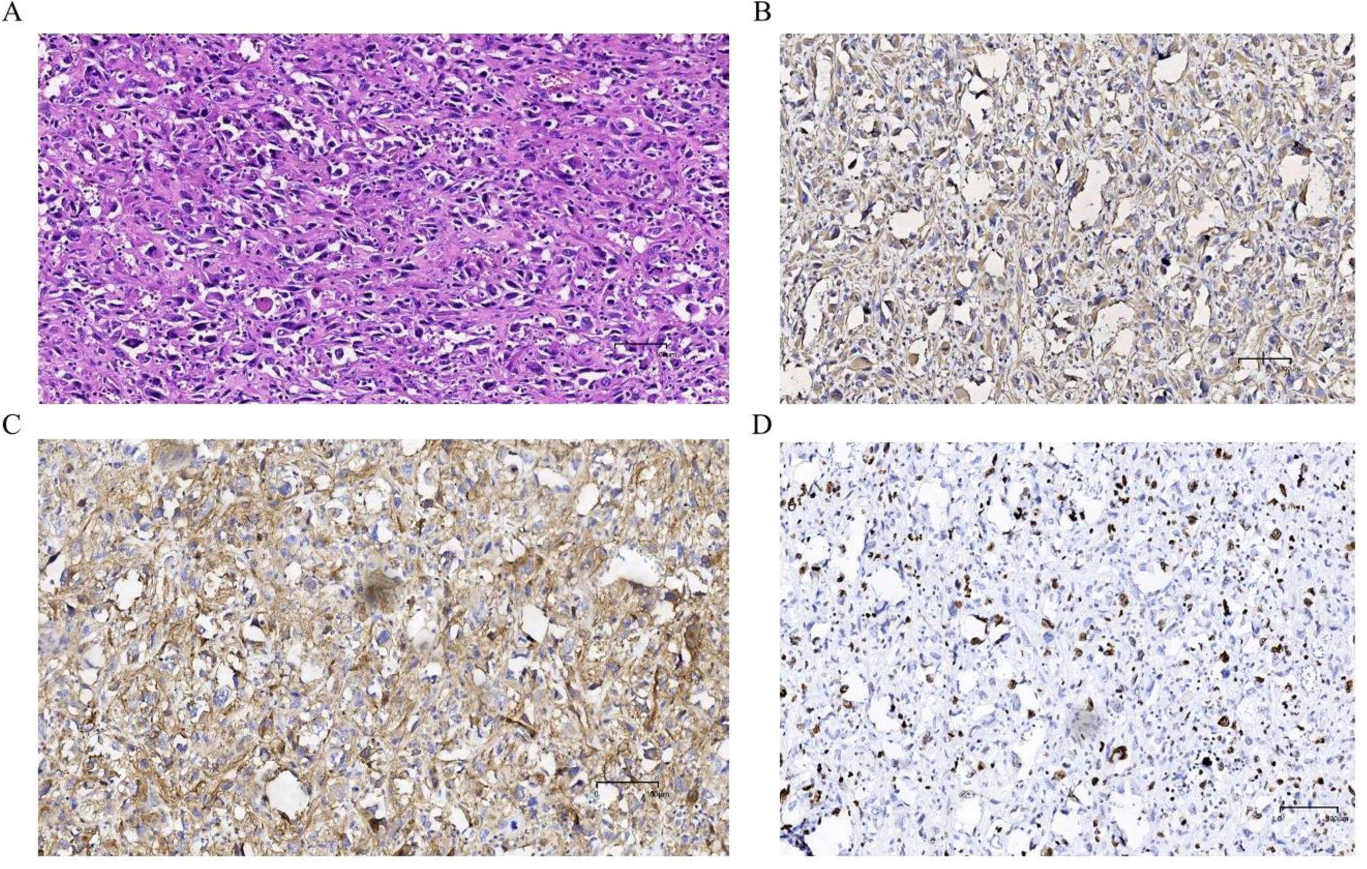
Postoperative HE staining and immunohistochemical examination: **(A)** The tumor tissue exhibits irregular arrangement, significant pleomorphism and polymorphism of tumor cells, with the presence of bizarre giant tumor cells and multinucleated tumor cells, mixed with varying numbers of spindle-shaped cells, and interstitial chronic inflammatory cell infiltration. Immunohistochemical examination: **(B)** Vimentin (+); **(C)** CD10 (+); **(D)** Ki67 (+, approximately 30%).

The postoperative pathological results indicated: Undifferentiated pleomorphic sarcoma of the left adrenal gland. Microscopic examination revealed: The tumor tissue was arranged irregularly, with significant atypia and pleomorphism of the tumor cells, presence of bizarre tumor giant cells and multinucleated tumor cells, interspersed with varying numbers of spindle-shaped cells, and chronic inflammatory cell infiltration in the stroma (**Figure 3A**); Immunohistochemical staining showed: Vimentin (+, **Figure 3B**), CD10 (+, **Figure 3C**), CD68 (+), Ki-67 (approximately 30%, **Figure 3D**). The patient recovered well postoperatively and was discharged 7 days after surgery. The patient was instructed to return to the hospital for chemotherapy one month later, with a chemotherapy regimen of: Dacarbazine 0.5g/m^2^ on days 1-3, Cyclophosphamide 0.8g/m^2^on day 1, Pirarubicin 50mg/m^2^ on day 1, with one week constituting one cycle, for a total of four cycles. During the chemotherapy process, the patient only experienced mild gastrointestinal symptoms, which were relieved after symptomatic treatment. Postoperative follow-up at 6 months showed no signs of tumor recurrence or metastasis.

## Discussion

UPS was first proposed and described by O’Brien et al. in 1964 (10). The incidence of UPS is higher in males than in females. It can theoretically occur in any part of the body but is most commonly seen in the extremities (with 50% occurring in the lower limbs and 20% in the upper limbs), and only a minority occur in the retroperitoneal space (11). The pathogenesis of this tumor is currently unclear, and previous studies have suggested that about 30% of undifferentiated pleomorphic sarcomas are genetically related, and 3%-5% are associated with radiation exposure (12, 13). Pathologically, UPS is a subtype of sarcoma that lacks specific differentiation, and the new edition of the WHO classifies it into three histological subtypes: pleomorphic, inflammatory, and giant cell types (14). However, these subtypes do not have a clear association with the prognosis of the disease (2). This tumor is a high-grade soft tissue sarcoma with a high degree of malignancy, with a 5-year recurrence rate ranging from 10% to 42%, and 31% to 40% will develop distant metastasis; its 5-year survival rate is also only between 30% and 50% (6). This case is a 44-year-old female with adrenal UPS, which is consistent with the age of onset reported in previous literature; UPS originating from the adrenal gland is extremely rare, and there is even less long-term follow-up data on adrenal UPS. After reviewing the English literature, this case is the first reported case of UPS originating from the adrenal gland.

Adrenal UPS often lacks specific clinical manifestations in its early stages, or it may only present with discomfort or pain in the waist and back. Despite being occasionally detected, it is difficult to determine its benign or malignant nature. Therefore, it is often diagnosed late, leading to delayed treatment. Currently, the detection of UPS before surgery mainly relies on CT scans. The following CT findings have some reference value: 1. Its tissue composition is complex, often appearing as a mixture of isodense and hypodense areas on CT; 2. In primary UPS lesions of retroperitoneal organs, mucinous components, hemorrhage, or necrosis can often be seen, which appear as heterogeneously enhancing soft tissue lesions on CT; whereas pleomorphic UPS, also shows metaplasia and calcification on CT, and its solid parts often exhibit peripheral moderate enhancement on enhanced CT, with the solid parts within the necrotic areas showing interspersed pseudopod-like or cotton-like changes, and enhancement occurs during both arterial and venous phases, characterized by a “fast-in and slow-out” pattern (15). In this case, the CT appearance of adrenal UPS showed a mixed density of isodense and hypodense areas, mainly dominated by soft tissue density, with irregular cystic areas within, which is considered to be the necrotic or hemorrhagic foci of the tumor; on enhanced CT, the solid parts showed significant heterogeneous enhancement. The CT imaging findings in this case are consistent with the literature reports. However, it is important to emphasize that the role of imaging in identifying diseases is limited. The gold standard for diagnosing this disease remains pathological examination, and imaging is usually only used for reference and auxiliary evaluation (16).

Adrenal UPS must be differentiated from pheochromocytoma and other adrenal tumors. Pheochromocytoma typically presents with the classic triad of symptoms: headache, palpitations, and sweating, along with paroxysmal or sustained hypertension; biochemical tests often show a significant increase in urinary or plasma catecholamines; on CT imaging, it appears as a homogeneous or heterogeneous solid or cystic mass, with areas of necrosis that may be accompanied by calcifications (17). In this case, the patient had a one-year history of hypertension, and the CT findings did not rule out the possibility of pheochromocytoma. However, the patient did not exhibit the typical clinical manifestations of elevated catecholamines, and upon admission, the patient’s serum cortisol, angiotensin II, renin, aldosterone, and aldosterone-renin ratio were all normal. Additionally, blood pressure monitoring after admission was within the normal range, thus allowing for a preliminary differentiation from pheochromocytoma. However, adrenal UPS is extremely rare in clinical practice, and soft tissue sarcomas are usually diagnosed by exclusion, as stated in this article. Most cases can only be definitively diagnosed after postoperative histopathological examination. Therefore, an accurate diagnosis of adrenal UPS still requires reliance on pathological examination.

The histopathological features of UPS are similar to those of general soft tissue sarcomas. Under light microscopy, they are primarily characterized by an indefinite arrangement, with marked pleomorphism of cells and nuclei, often accompanied by bizarre tumor giant cells, multinucleated tumor cells, osteoclast-like giant cells, and mixed with varying numbers of spindle-shaped cells and round histiocytic-like cells, showing active mitotic activity, with or without tumor necrosis. Different pathological types of UPS exhibit certain characteristics: the giant cell type UPS presents with typical mononuclear or multinucleated tumor giant cells; whereas the inflammatory type UPS has a significant infiltration of inflammatory-like cells (18). In this case, the postoperative pathology showed: adrenal UPS. Microscopically, the tumor tissue showed an indefinite arrangement, with significant atypia and pleomorphism of tumor cells, presence of bizarre tumor giant cells and multinucleated tumor cells, mixed with varying numbers of spindle-shaped cells, and chronic inflammatory cell infiltration in the stroma. These findings are basically consistent with the pathological characteristics of UPS reported in the literature. The immunohistochemical results of this case showed: Vimentin(+), CD10(+), CD68(+), Ki-67(+, approximately 30%), all positive. This is consistent with the UPS immunohistochemical results reported previously (19). It should be noted that UPS is a diagnosis of exclusion, and in pathological diagnosis, it must also be differentiated from tumors such as synovial sarcoma, high-grade myxofibrosarcoma, and others (20).

UPS have been reported in various sites, including the pharynx (3), kidney (4), spleen (5), and pancreas (21). Among these, UPS of the pharynx, spleen, and kidney have been associated with survival periods of 5 years, 6 months, and 10 years, respectively, following radical surgical resection. In contrast, UPS of the pancreas developed pulmonary lymph node metastasis within 7 months after tumor resection and resulted in death 5 months postoperatively. Compared to these cases, to date, the patient in this case has shown no signs of tumor recurrence or metastasis for half a year. This may be attributed, on one hand, to early diagnosis and radical resection of the tumor at an early stage. On the other hand, postoperative adjuvant chemotherapy may also play a positive role. We will continue to follow up on this case to provide some reference. Concurrently, immuno-targeted therapy for UPS is under active investigation, which may open up new directions for the treatment of UPS (22).

## Conclusion

In summary, primary adrenal UPS is an extremely rare tumor. Although adrenal UPS does not have obvious clinical manifestations, clinicians should be vigilant about the possibility of adrenal UPS when encountering adrenal masses of unknown nature. Currently, radical tumor resection remains the most effective treatment method, and adjuvant chemotherapy postoperatively can improve the survival rate of patients. Of course, this requires more clinical cases for validation and long-term follow-up studies to understand the exact biological behavior of the tumor. This case emphasizes the clinical pathology, imaging, and other manifestations of adrenal UPS, and we will continue to follow up on this case to understand the prognosis of the disease, with the aim of providing a reference for the treatment of primary adrenal UPS.

## Data Availability

The original contributions presented in the study are included in the article/**Supplementary Material**. Further inquiries can be directed to the corresponding author.
