# Supplementary figures and images for: Undifferentiated pleomorphic sarcoma of the adrenal gland: a case report and literature review
Source: Front Oncol. 2024 Nov 18;14:1439357. doi: 10.3389/fonc.2024.1439357 (PMC11609073; doi:10.3389/fonc.2024.1439357)

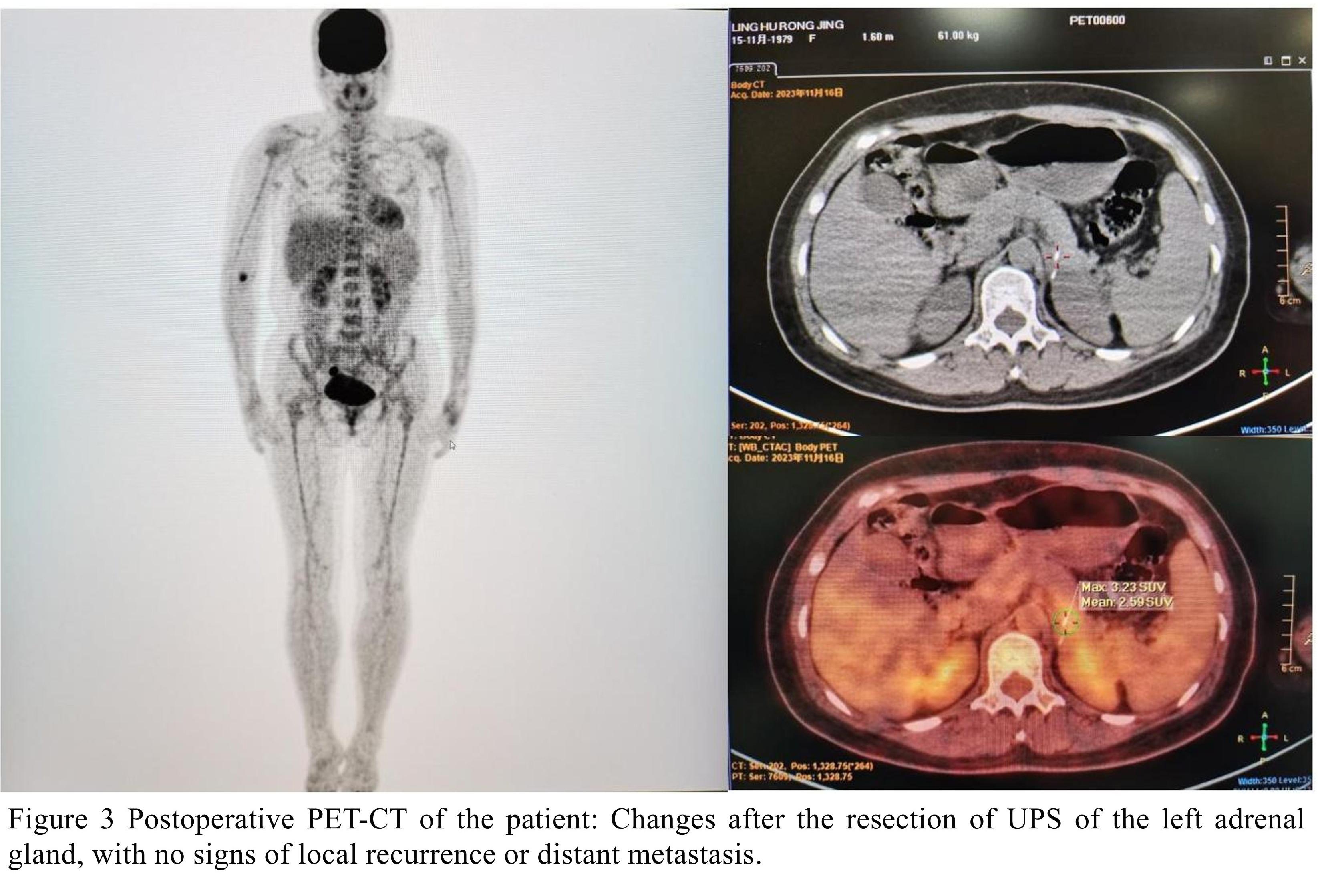

Supplement: Supplementary file 1 [file Image1.jpeg]
